# A pilot study investigating the effects of a manuka honey sinus rinse compared to a standard sinus rinse on sino-nasal outcome test scores in cystic fibrosis patients

**DOI:** 10.1186/s40814-022-01175-0

**Published:** 2022-09-24

**Authors:** Aled E. L. Roberts, Cendl Xanthe, Alison L. Hopkins, Owen Bodger, Paul Lewis, Eshwar Mahenthiralingam, Jamie Duckers, Rowena E. Jenkins

**Affiliations:** 1 Swansea University Medical School, Swansea University, Swansea, SA2 8PP Wales; 2 All Wales Adult Cystic Fibrosis Centre, Llandough Hospital, Penarth, CF62 2XX Wales; 3 Microbiomes, Microbes and Informatics group, Organisms and Environment Division, Cardiff School of Biosciences, Cardiff University, Cardiff, CF10 3AX Wales

**Keywords:** Cystic fibrosis, Respiratory infections, Infection control, Microbiology, Therapeutics

## Abstract

**Background:**

People with cystic fibrosis (CF) are prone to bacterial respiratory infections; these are often antibiotic resistant, are difficult to treat, and impact on the quality of life and lung function. The upper respiratory tract can act as a reservoir for these pathogens, and as part of clinical care, sinus rinses are used to alleviate symptoms in the upper airway. We have developed a sinus rinse containing manuka honey, to identify whether it can help improve symptoms or reduce the bacterial load.

**Methods:**

We will undertake a randomised controlled trial where 30 adults with CF will be recruited and randomised to either the control or intervention group. Both groups will follow a sinus rinse protocol for 30 days (± 7 days); the control group will use the standard of care rinse, and the intervention group will use a manuka honey rinse. Both groups will provide samples at day 0 and day 30. The primary outcome measure will be a change in the 22-item Sino-Nasal Outcome Test (SNOT-22) score. Secondary outcomes will include changes to quality of life (questionnaire), bacterial load/community composition, and sputum viscosity.

**Discussion:**

This trial will look at the use of a manuka honey-infused sinus rinse solution on patients diagnosed with cystic fibrosis (CF) suffering with sinusitis; it will allow us to determine the efficacy of the manuka honey sinus rinse compared to standard rinse and will allow us to determine if molecular bacterial diversity analysis will provide in-depth information beyond the usual conventional microbiological. It will allow us to determine the feasibility of recruiting participants to this type of trial, allow us to check participant compliance with the protocol, and inform future studies.

**Trial registration:**

Approval was obtained from the Research Ethics Committee Wales REC7 reference 18/WA/0319. Results of this study will be published at international conferences and in peer-reviewed journals; they will also be presented to the relevant stakeholders and research networks.

Trial registration number: ClinicalTrials.gov Identifier NCT04589897 (retrospectively registered)

## Background

Cystic fibrosis (CF) is a one of the most common autosomal genetic disorders affecting western populations [1]. Multiple mutations in the cystic fibrosis transmembrane conductance regulator gene (*cftr*) are associated with the disease with many leading to the loss of functional cystic fibrosis transmembrane conductance regulator (CFTR) proteins in epithelial membranes [2]. These CFTR chloride channels are critical to maintaining a healthy salt-water balance across epithelial membranes [3]. Loss of function in the epithelial surfaces of CF patients results in dehydration of surface mucus and a build-up that severely affects muco-ciliary clearance [4, 5]. The thick and sticky mucus is capable of acting as a rich nutrient source for opportunistic pathogens, promoting infections from a wide range of bacteria species such as *Staphylococcus aureus*, *Pseudomonas aeruginosa*, *Burkholderia cepacia* complex species, *Haemophilus influenzae*, and *Stenotrophomonas maltiphilia* [6].

Once certain bacteria infect the CF lung, they are very difficult to remove. It is clear that certain bacterial species become more prevalent pathogens at different times of life [6]. In the early stages of life (< 18 years old), *S. aureus* is the most prevalent infection, accounting for half of all CF respiratory disease cases. As lung damage and chronic infections progress over time, *P. aeruginosa* becomes a dominant infective species, accounting for ~41.4% of all UK adult CF lung infections (UK CF Patient Registry) [7–10].

Currently, patients are prescribed antibiotics to control these bacterial infections, with many patients on long-term oral, intravenous, and nebulised antibiotic treatment [11]. This type of long-term exposure to antibiotics leads to the emergence of antimicrobial resistance (AMR) amongst bacterial cells, which is now recognised globally as one of the major threats to human health. Globally, 700,000 people die each year as a result of AMR with far-reaching effects, resulting in an inability to control infections, increased morbidity and mortality, decreased patient wellbeing, and an overall increase in associated healthcare costs [12]. The notion of AMR is nothing new to the majority of CF patients whom through the prolonged use of antibiotics have experienced this first-hand already [13].

One of the key problems with CF-lung infections is the frequency of re-infection from various infection reservoirs [14]. The upper respiratory tracts (upper airways and sinuses) of CF patients are frequently infected and therefore act as major reservoirs of infection for the lower respiratory tract [15]. Therefore, complete clearance of lung infections is not readily possible due to the potential for reinfection. Thus, lung transplants are limited to patients where the infection reservoir can be controlled effectively, as failure to do so will result in re-colonisation of the new lungs and transplant failure [16]. For instance, patients colonised with *Burkholderia cepacia* complex (BCC) species have worse survival rates post transplantation compared to BCC-negative patients [17]. Therefore, we have an urgent need for new and sustainable antimicrobial treatments for those suffering with CF as over 80% of patients will require treatment as a result of chronic infection and subsequent inflammation which ultimately leads to lethal respiratory failure [10].

For many people with CF, antibiotic therapy is ineffective due to the infecting strain/species having extensive AMR mechanisms [18, 19]. If this is coupled with a hypervirulent strain or species, then a rapid decline in lung function can follow to the point where it becomes fatal [20, 21]. The sino-nasal cavity is one of the main infection reservoirs for the lungs as mucus and debris are breathed into the lungs [15]. If there is any potential to change the bacterial load of the sino-nasal cavity (so that antibiotics work again) or kill hyper virulent pathogens, then the number of treatment options available to patients would increase favourably.

Manuka honey has a strong track record as an antimicrobial agent, inhibiting a wide range of opportunistic pathogens, many of which cause problematic chronic infections and have the ability to colonise the airway of CF patients [22, 23]. Further to this, AMR towards manuka honey has not been observed [24]. By combining manuka honey with a nasal rinse procedure, we predict that the upper nasal cavity could be “decolonised” of chronic pathogens.

The objective of this pilot study is to determine the feasibility of recruiting participants to this type of trial, allowing us to check participant compliance with the protocol to determine the effect of a manuka honey sinus rinse on the symptoms associated with sino-nasal disorders, along with any corresponding quality of life improvements. Furthermore, to gain additional information, we will measure the effects of manuka honey on bacterial load and community composition in various samples associated with the nasal cavity and the CF-lung. The results will help facilitate the design and feasibility of a multicentred study into the use of a manuka honey sinus rinse to alleviate symptoms in those with poor sino-nasal scores. Understanding beneficial changes in community composition could also help design and infer studies into manuka honey as a potential nebulised product.

## Methods/design

This is a single-blinded randomised feasibility study to estimate the efficacy of a manuka honey sinus rinse on symptoms associated with sino-nasal disorders in CF patients. Recruitment will take place at the All Wales Adult Cystic Fibrosis Centre (AWACFC) at University Hospital Llandough (Cardiff & Vale University Health Board) over an 18-month period, starting January 2019 and ending October 2021 (study paused March 2020–July 2021 due to COVID). This trial will help to determine the potential of manuka honey as an adjuvant for current sinus rinse protocols designed for CF patients. It will also allow us to establish manuka honey’s baseline in vivo antibacterial efficacy and the changes this may have on microbial community composition so that it can be developed further to aid the removal of problematic strains/species. The SPIRIT reporting guidelines have been used for this manuscript [25].

### Aims

#### Feasibility aims

- The primary aim of this trial is to determine its feasibility (patient recruitment and compliance with the intervention strategies).

#### Primary clinical aim

- The primary clinical objective of this trial is to determine whether the addition of manuka honey to a sinus rinse impacts on the SNOT-22 score of patients with CF.

#### Secondary clinical aims

- Determine if the addition of manuka honey to the sinus rinse alters the quality of life for patients with CF
- Establish if the addition of manuka honey to the sinus rinse impacts upon bacterial load and/or community composition in the nasal/paranasal cavities of patients with CF
- Determine if the addition of manuka honey to the sinus rinse alters the structure/viscosity of sputum mucus in patients with CF

### Trial design

The study (Protocol v2.0) will be structured as a 2-sided equality trial, with before and after measurements for each participant. During the 18-month study period, we aim to sample approximately 30–40 patients. This is a proposed sample size that combines a power calculation and a consideration for meeting the eligibility criteria and drop-out rates. We expect to screen 290 patients per year from a single CF centre (AWACFC), of which 50% (145) will complete a SNOT-22 questionnaire. Approximately 50% (72) of those completing the SNOT-22 questionnaire should be within our eligibility criteria. If we allow for a conservative recruitment rate (30–40%), it would be realistic to enrol 20–30 patients per year. In order to obtain a power of 80%, we would require a sample size of at least 21. We therefore propose that a target sample size of 30, since this will be both achievable and will deliver sufficient power, even allowing for potential patient drop-out.

Eligible patients will be randomly assigned into one of two groups with an allocation ratio of 1:1, split into three consecutive groups of ten patients (to ensure even distribution of groups 1 and 2 throughout the study). Group 1 is the control group receiving the standard nasal rinse protocol and will be assigned 50% of the participants. Group 2 is the intervention arm on a manuka honey sinus rinse protocol and will be assigned 50% of the participants.

### Control arm: group 1

Participants randomly assigned to group 1 will receive a nasal rinse protocol to be completed daily for 30 days (± 7 days) that is in line with current gold standard treatment.

### Intervention arm: group 2

Participants randomly assigned to group 2 will receive a modified nasal rinse protocol containing manuka honey to be completed daily for 30 days (± 7 days).

### Endpoints

The sampling will conclude on the date of the last visit of the last participant undergoing the trial or once an adequate number of participants have completed the study so that the power calculation can be satisfied.

### Participant selection

Potential participants attending the AWACFC will be initially identified by members of the clinical research team (that also form part of the patient’s standard clinical care team). Written patient information sheets will be provided to potential participants who will be given a minimum of 24 h to consider enrolling on the study. The study will then be fully explained to the patient, and if they are satisfied with the information they have received, meet the inclusion/exclusion criteria, and provide written informed consent, they will be enrolled onto the study (Table 1).

**Table 1:** Participant selection criteria

Inclusion criteria	Exclusion criteria
• The patient is willing and able to give informed consent • The patient must be ≥ 18 years • The patient must have an established diagnosis of CF (one or more of the following), sweat chloride > 60 mEq/L and/or presence of two CF-causing mutations • The patient must have chronic symptoms of rhinosinusitis according to the criteria of the European Position Paper on Rhinosinusitis • The patient scores greater ≥ 7 on their SNOT-22 questionnaire	• The patient has ever tested positive for the bacteria *Mycobacterium tuberculosis* • The patient is currently using a nasal rinse protocol • The patient has undergone sinus surgery within 6 months • The patient suffers from nasal bleeding • The patient is currently undergoing systemic antibiotic therapy for infective exacerbation • The patient is using overnight oxygen via nasal cannula • The patient is participating in another clinical trial or has done so within the last 30 days • The patient has a known allergy to bee products

### Interventions and investigational products

The investigational product is a manuka honey sinus rinse. Manuka honey is a CE-approved medical device commonly used for the treatment of surface wound infections. This study will use a standard nasal rinse protocol for CF patients with/without manuka honey (Medihoney, DermaSciences, UK). Both control and intervention groups will make up a sinus rinse solution comprised of non-iodized salt (½ teaspoon), bicarbonate of soda (½ teaspoon), and filtered water (up to ½ pint); however, intervention groups will add in manuka honey (2× 50 g tubes). Participants will do this daily for a total of 30 days (± 7 days), keeping a compliance diary and taking any observational notes to help determine compliance with the protocol.

### Study setting

Recruitment of participants will solely take place at the All Wales Adult Cystic Fibrosis Centre (AWACFC) at University Hospital Llandough with sample processing and data analysis split between the Cardiff School of Biosciences, Cardiff University, and the Institute of Life Sciences, Swansea University. The study sponsor is Swansea University, Swansea, SA2 8PP, UK, email:paola.a.griffiths@swansea.ac.uk

### Randomisation

Study participants will be randomly assigned into either the control or intervention arm of the study using the GraphPad Quick Calcs random number generator. To ensure even distribution into both control and intervention arms of the study, a 50:50 split for every 10 participants enrolled onto the study will be employed.

### Blinding

Academic research staff analysing all of the data will be blinded to the group allocation. Only the clinical research team will remain unblinded throughout the study. Unblinding will occur once data collection and sample processing has been completed.

### Participant timeline and schedule of assessment

**Fig. 1:**
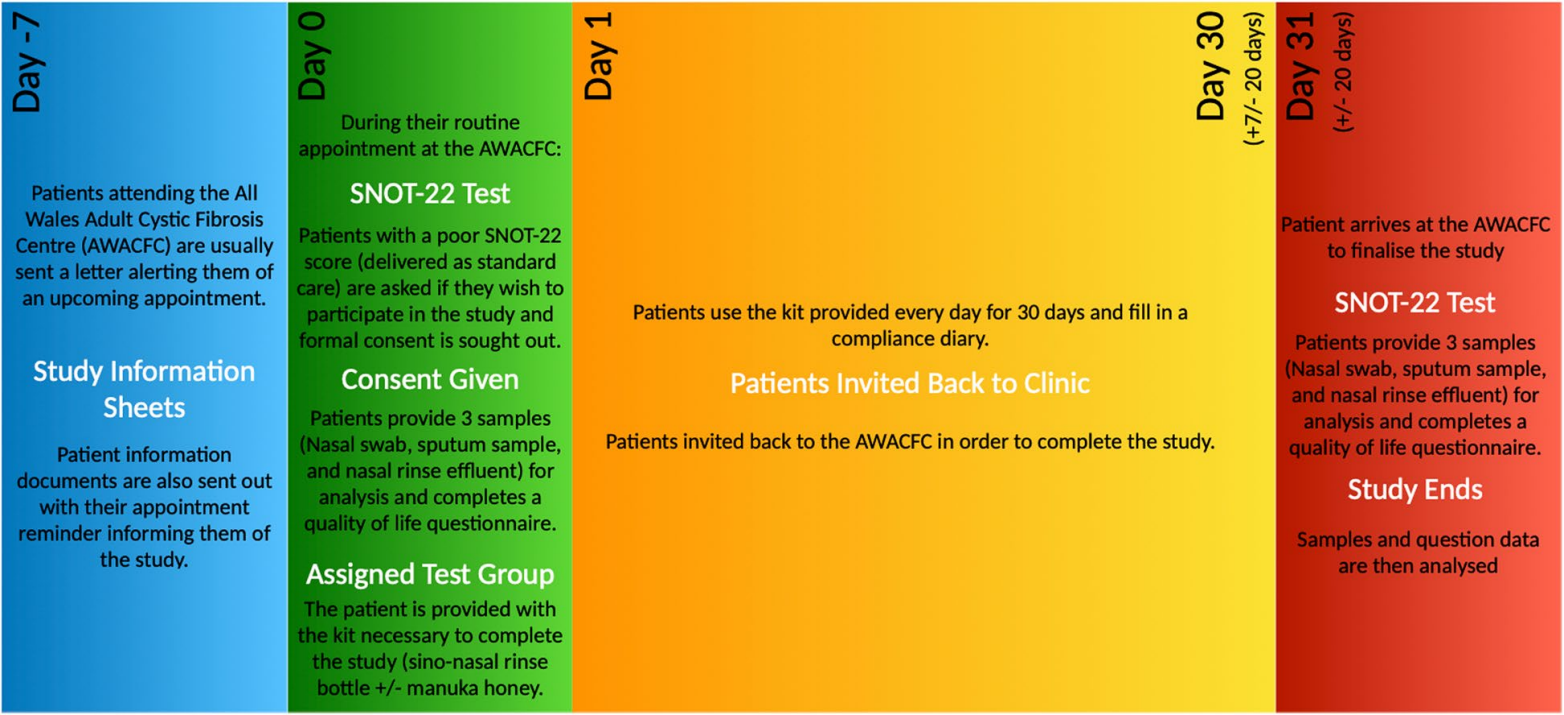
Flowchart showing study timeline

Figure 1 shows the timeline of events from pre-participant recruitment to participant completion. Prior to the study, potential participants will be approached and supplied with information pertaining to the study so that they can make an informed decision as to whether to take part. On day 0 as part of baseline measurements, participants will undergo assessment for eligibility in the study using validated tools (SNOT-22 questionnaire) alongside quality of life questionnaires (CFQ-R). Baseline samples to determine bacterial load and community composition will be taken (nasal swab, sputum, and sinal rinse effluent samples) and processed. Bacterial load will be determined using conventional microbiological techniques and next-generation sequencing of the bacterial 16S rRNA gene [26]. Participants will then undergo treatment as part of either the control or intervention arm of the study. After 30 days (± 7 days) at a follow-up meeting, repeat measurements will be taken. Patients will be encouraged to complete the compliance diary, noting any symptoms/discomforts that arise during the 30 day (± 7 day) testing period.

### Withdrawal

Participants of the study have the right to withdraw at any time. It may be necessary for the clinical care team to discontinue the participant from the study at any time should they deem it necessary. The reason for withdrawal (if supplied) will be recorded on the clinical record form. Should time allow, new participants will be recruited onto the study to replace those that have withdrawn. If a patient withdraws from the study, all identifiable samples will be destroyed; however, all the data generated up to the date of the withdrawal will remain for analytical purposes.

### Data collection and management

Baseline data collection will start on day 0 when participants are enrolled onto the trial, with additional repeat measurements taken during the participant’s follow-up meeting (day 30 ± 7 days). Non-identifiable clinical data recorded as part of standard clinical practice will be entered onto the CF registry and is kept indefinitely as part of standard clinical practice; additionally, it will be recorded on the clinical record form (CRF) using the participant’s unique identifier. Data will be stored on a computer database that contains no identifiable information, to be analysed once the final participant has completed the study.

The SNOT-22 questionnaire will be used to determine the severity of social-emotional consequences of a participant’s nasal disorder and reflects the feelings of patients towards their nasal disorder (some areas covered in the questionnaire are problems relating to sneezing, runny nose, dizziness, nasal discharge, fatigue, etc.). The CFQ-R questionnaire determines the impact of CF on a patient’s life so that treatment adjustments can be made to improve their quality of life. Some of the areas surveyed relate to; demographics, quality of life, school/work/daily life activities, and symptom difficulties.

### Conventional microbial culturing

We will use a range of selective microbiological agar (such as mannitol salt, *Pseudomonas* isolation, and *Burkholderia cepacia* selective agar) to determine the bacterial load (of specific bacteria, such as; *Staphylococcus*, *Pseudomonas*, and *Burkholderia* species) in each clinical sample (nasal swab, sputum, and sinal rinse effluent).

### DNA extraction and whole genome sequencing

DNA will be extracted using the Maxwell 16 Tissue DNA Purification Kit (Promega) and robotic Maxwell 16 System (Promega). The total sputum DNA will then be subjected to 16S rRNA gene sequencing to determine the bacterial diversity in the samples. This will be performed at the Cardiff University Genomics Research Hub by amplifying the V4 region of the 16S rRNA gene and sequencing these PCR products using the Illumina MiSeq platform (Illumina). Controls for DNA extraction as well as a DNA mock bacterial community will be included.

### Sputum analysis

Fourier transform infrared spectroscopy will be used to determine if the manuka honey rinse interacts with sputum mucus (secreted mucin glycoprotein) and characterise any molecular structural changes.

### Participant rights and confidentiality

The chief investigator will ensure all study data is kept confidential and managed in accordance with General Data Protection Regulation, the Research Governance Framework, and the awarding Research Ethics Committee. Data will be maintained on a password-protected database.

### Statistical analysis

Recruitment numbers will be assessed at the end of the study to see if they match the number predicted in the initial power calculation. The compliance diaries will be checked to determine adherence to the study protocols. Qualitative analysis of SNOT-22 and quality of life questionnaires (CFQ-R) scores will be conducted between control and treatment arms of the study as well as before and after treatment. To determine if manuka honey has had an effect on bacterial load and/or bacterial community composition, a contingency table with odds ratio will be prepared taking into account co-variants.

### Safety measures

An adverse event (AE) is defined as any untoward medical occurrence within the trial (even if there is no obvious causal relationship with the experimental treatments). An AE will therefore be classed as any unfavourable and/or unintended sign, symptom, or disease occurring within the trial. Signs of potential allergic reaction (such as rashes, hives, itching, red and/or swollen skin, blistering and/or peeling skin, wheezing, tightness in the chest or throat, trouble breathing/swallowing/talking, unusual hoarseness, swelling of the mouth/face/lips/tongue/throat or nasal irritation) will be recorded. Various AEs may be expected with routine use of a sinus rinse protocol (such as nasal congestion, ear discomfort, nasal drainage post use, and accidental ingestion of solution) and will be recorded. A serious adverse event (SAE) is defined as any AE that results in death, is life-threatening, results in hospitalisation (or prolongs existing hospitalisation), results in persistent/significant disability and/or incapacity, results in congenital anomaly and/or birth defect, or results in any other medically important condition. In the event of a SAE, the sponsor and the AWACFC will be notified within 24 h and will inform the relevant Research Ethics Committee within 15 days using the National Research Ethics Service (NRES) report for SAEs. Unrelated and expected SAEs do not require reporting, but a copy of the SAE report will be retained in the study master file for audit purposes by research governance.

### Patient sampling

One of several respiratory consultants or clinical research nurses from the All Wales Adult CF Clinic, Llandough Hospital, Cardiff and Vale University Health Board, will collect all samples (swab, sputum and sinus rinse) at day 0 and on return visit. The clinical team will ensure that the academic team performing the microbiology and statistical analysis are blinded to the intervention that the patient has received.

### Management group

Monitoring visits to the site will be made every 3 months during the study by the trial management group to ensure that all aspects of the protocol are followed. The monitoring visit timeframe can be changed depending on the monitoring findings. A quality assurance programme is also in place to ensure adherence to the study protocol and any deviations from the protocol will be submitted to the ethics committee before being implemented.

### Patient involvement

Information sent to prospective participants of the study will be critiqued by selected patients at the AWACFC to ensure the information within is clear, concise, and understandable.

### Ethics and dissemination

Ethics approval was given by Research Ethics Committee Wales REC7 reference 18/WA/0319. The chief investigator of the study will ensure all those involved in the running of the study comply with the applicable regulatory requirements (including but not limited to the Research Governance Framework along with university, hospital and sponsor research office policies). Written informed consent (completed in accordance with good clinical practice) will be obtained from all participants by the clinical research team. Any SAEs occurring within the study will be reported to the relevant committees.

Results from the trial will be disseminated through oral and poster presentations at national conferences, through peer-reviewed journal publications, via leaflets/posters at the AWACFC, and in video format online by named members of the trial management group and those involved with sample preparation, data analysis, and manuscript preparation. Annual reviews will be submitted to the relevant bodies to ensure the trial is progressing as per the original design. Participants will be supplied with leaflets detailing the main findings of the study.

## Discussion

At present, the number of studies looking into the efficacy of sinal rinse protocols on SNOT-22 scores is limited. The primary function of a sinal rinse is to alleviate symptoms associated with sino-nasal disorders which includes the removal of mucus, allergens, and bacteria, reducing the overall inflammation of the nasal cavity [27]. Manuka honey is known to have excellent broad-spectrum antimicrobial activity; however, much of the current studies have revolved around its use in surface wounds [28–31]. Few studies have looked at the effects of manuka honey with regards to respiratory infections, focussing on chronic rhinosinusitis instead of CF specifically [26, 32]. These studies both show decreased levels of microbial load in manuka honey-treated patients. The broad-spectrum antimicrobial activity of manuka honey could prove useful, inhibiting multiple pathogens colonising the CF airway. This study will probe the feasibility recruiting participants and their compleience to the study protocols of the trial and will help guide the main study as comparative protocols are limited. It is thought that the current design allows for robust testing of protocols whilst providing transparency on the methods used. Successful feasibility would be recruitment that matched the initial numbers predicted by the power calculation allowing for the predicted dropout and non-complicance. The primary clinical goal of this study is to determine the effects on a patient’s quality of life metrics; however, as many of these are linked to the underlying infective status, it is thought that alteration of bacterial load/diversity will have an effect on these metrics. It is hoped that alterations in bacterial load/diversity (in line with previous chronic rhinosinusitis studies) within the sino-nasal cavity (which acts as a reservoir of infection) may also alleviate symptoms associated with CF. If changes are observed within the sino-nasal cavity and pathogenic culture load decreases, then it might be possible to clear harmful infection reservoirs prior to lung transplantation. Alternatively, alterations in diversity may prove prudent if virulent strains/species are inhibited. It is thought that through the methodology used in this study, we can characterise the effects sinus irrigation (with and without manuka honey) has on various aspects of infection in CF patients. It is thought the completion of this feasibility study will inform best practice, whilst allowing unearthed uncertainties in the study design to be addressed prior to completing the main study.

## Data Availability

Results from the trial will be disseminated through oral and poster presentations and national conferences and through peer-reviewed journal publications.

## Abbreviations

AE: Adverse events
AMR: Antimicrobial resistance
AWACFC: All Wales Adult Cystic Fibrosis Centre
CFQ-R: Cystic Fibrosis Questionnaire-Revised
CI: Chief investigator
CRF: Case report form
ECFSPR: European Cystic Fibrosis Society Patient Registry
FEV1%: Forced Expiratory Volume (in 1 second as percentage of predicted)
GCP: Good clinical practice
GP: General practitioner
HCRW: Health and Care Research Wales HRA Health Research Authority
HTA: Human Tissue Authority
NICE: National Institute for Health and Care Excellence NHS National Health Service
MHRA: Medicines and Healthcare Products Regulatory Agency
NRES: National Research Ethics Service
PI: Principal investigator
PIS: Participant information sheet
R&D: Research and development
REC: Research Ethics Committee
SAE: Serious adverse events
